# Assessing the quality of evidence on safety: specifications for application and suggestions for adaptions of the GRADE-criteria in the context of preparing a list of potentially inappropriate medications for older adults

**DOI:** 10.1186/s12874-022-01715-5

**Published:** 2022-08-30

**Authors:** Tim Mathes, Nina-Kristin Mann, Petra Thürmann, Andreas Sönnichsen, Dawid Pieper

**Affiliations:** 1 Department of Clinical Pharmacology, School of Medicine, Faculty of Health, Witten/Herdecke University, Witten, Germany; 2 Department of Medical Statistics, University Medical Center Göttingen, Göttingen, Germany; 3 Institute for Research in Operative Medicine, School of Medicine, Faculty of Health, Witten/Herdecke University, Cologne, Germany; 4 Philipp Klee-Institute for Clinical Pharmacology, Helios University Hospital Wuppertal, Wuppertal, Germany; 5 Institut für Wissensmanagement in der Medizin, Salzburg, Österreich; 6 Faculty of Health Sciences Brandenburg, Brandenburg Medical School Theodor Fontane, Institute for Health Services and Health System Research, Neuruppin, Germany; 7 Center for Health Services Research, Brandenburg Medical School Theodor Fontane, Neuruppin, Germany

**Keywords:** Evidence synthesizes, systematic reviews, potentially inadequate medications, PIM-List, GRADE, Level of Evidence, safety, harms

## Abstract

**Background:**

Systematic reviews that synthesize safety outcomes pose challenges (e.g. rare events), which raise questions for grading the strength of the body of evidence. This is maybe one reason why in many potentially inappropriate medication (PIM) lists the recommendations are not based on formalized systems for assessing the quality of the body of evidence such as GRADE.

In this contribution, we describe specifications and suggest adaptions of the GRADE system for grading the quality of evidence on safety outcomes, which were developed in the context of preparing a PIM-list, namely PRISCUS.

**Methods:**

We systematically assessed each of the five GRADE domains for rating-down (study limitations, imprecision, inconsistency, indirectness, publication bias) and the criteria for rating-up, considering if special considerations or revisions of the original approach were indicated. The result was gathered in a written document and discussed in a group-meeting of five members with various background until consensus. Subsequently, we performed a proof-of-concept application using a convenience sample of systematic reviews and applied the approach to systematic reviews on 19 different clinical questions.

**Results:**

We describe specifications and suggest adaptions for the criteria “study limitations”, imprecision, “publication bias” and “rating-up for large effect”. In addition, we suggest a new criterion to account for data from subgroup-analyses. The proof-of-concept application did not reveal a need for further revision and thus we used the approach for the systematic reviews that were prepared for the PRISCUS-list.

We assessed 51 outcomes. Each of the proposed adaptions was applied. There were neither an excessive number of low and very low ratings, nor an excessive number of high ratings, but the different methodological quality of the safety outcomes appeared to be well reflected.

**Conclusion:**

The suggestions appear to have the potential to overcome some of the challenges when grading the methodological quality of harms and thus may be helpful for producers of evidence syntheses considering safety.

**Supplementary Information:**

The online version contains supplementary material available at 10.1186/s12874-022-01715-5.

## Background

Clinical practice recommendations (for example in guidelines) that are based on the best available evidence can improve quality of care [1, 2]. Lists of potentially inappropriate medication (PIM) name drugs, which may have a negative risk-benefit-ratio in older patients, especially when safer alternatives are available [3, 4]. A variety of PIM-lists have been prepared or adapted to the local drug market in different countries. One reason for establishing PIM-lists on expert ratings is the frequent exclusion or low inclusion rate of older patients, particularly frail older people in clinical trials, which represent the basis for drug approval as well as the evidence in clinical practice guidelines. However, an often-discussed limitation of existing PIM-lists is that they are not based on systematic reviews of the evidence, but only on expert-opinion, unsystematic literature reviews or previously published PIM-lists [3]. This might be one reason why the overlap between PIM-lists is often low [3]. To overcome this limitation, for the update of the German PIM-list, namely the PRISCUS list, the participating experts were provided data from systematic reviews conducted specifically to inform the recommendations.

To grade the quality/levels of evidence for practice recommendations grading systems have been developed [5, 6]. The Grading of Recommendations Assessment, Development and Evaluation (short GRADE) system is one of the most established tools for rating the quality of evidence underlying recommendation in clinical practice guidelines [6]. GRADE rates the quality of evidence for a specific outcome across the included studies on a PICO (participants, intervention, comparison, outcome)-question. For this purpose, explicit criteria are used. These include the study design, study limitations, imprecision, inconsistency, indirectness, dose-response association and magnitude of effect. Based on these criteria the quality of evidence is classified into four levels (high, moderate, low or very low). Noticeably, in contrast to most other approaches which classify the strength of evidence on study level (e.g. randomized controlled trials [RCTs] are high and case reports are low level of evidence), GRADE rates the evidence for each outcome across the included studies.

Usually, the evidence is weaker for safety than for effectiveness outcomes because of inconsistent measurement, imprecision (studies are not powered for safety outcomes, rare events) and poor reporting of harms [7, 8]. Other challenges include that safety outcomes are often rare, unpredictable or require very long follow-up times to be detected. Furthermore, harms are often sub-group specific, but the relevant groups, such as frail older people are underrepresented in RCTs [9, 10]. For these reasons, safety may not have been sufficiently assessed in randomized controlled trials (RCTs). Consequently, only including RCTs in systematic reviews considering safety might not be sufficient. To generate sufficient evidence on safety it is advisable to include non-randomized studies (NRS) and to consider sub-groups analyses [11]. Therefore, considering other aspects of the quality of evidence in addition to risk of bias, in particular considering random error appears even more important for harms than for effectiveness.

One might argue that the quality of evidence on safety is just the way it is and no specific rating criteria for quality of evidence are necessary. However, if rating proceeds in the standard way some problems may come up. First, it bears the risk that the certainty of evidence between benefit and harms is unbalanced per se because benefits tend to get higher methodological quality ratings (e.g. because only NRS are available for a very rare harm). Second, there may be the methodological problem that a difference in the quality of evidence on different safety outcomes could not be differentiated because of floor effects, i.e. all studies are classified as low or very low quality of evidence. This is because in the GRADE system evidence from NRS always starts at low quality and thus only one additional criterion for rating down (e.g. imprecision) would result in a very low quality of evidence rating [6].

Some older evidence level classifications schemes, such as the oxford level of evidence use different criteria for harms and benefits, but there is no specific GRADE guidance for assessing the quality of evidence for safety outcomes [5].

In this contribution, we describe specifications and adaptions of the GRADE system for grading the quality of a body of evidence on safety outcomes and report our first experience of applying these for the development of a PIM-list.

## Methods

This project was part of the update of a PIM-list for older people in Germany, namely the PRISCUS list [12]. GRADE is the most established tool to assess the quality of evidence. We decided to specify its application and suggest potential adaptions instead of developing our own criteria for the following reasons. First, it can be assumed that in general the criteria relevant for assessing the quality of evidence on harms are (almost) the same as for benefits. Second, it facilitates the integration of evidence on benefits and harms within one evidence synthesis product (e.g. clinical practice guideline). Safety outcomes are mostly binary outcomes or expressed as such (e.g. patients suffering anemia instead of hemoglobin in g/L below normal). Furthermore, the challenges when summarizing harms are more prominent for binary outcomes (e.g. rare events). Therefore, we only consider binary variables in this work.

### Development of specifications and adaptions

The research team consisted of five members; two experienced methodologists, one senior clinical pharmacologist, one senior general practitioner, and one pharmacist. The members assessed each of the five GRADE domains for rating down (study limitations, imprecision, inconsistency, indirectness, publication bias) and the criteria for rating up, considering whether special considerations or revisions of the original GRADE approach were indicated. We judged a specification or adaption as indicated, if it could be expected that one of the challenges quoted in the introduction affect the original GRADE criteria (e.g. higher imprecision, inclusion of NRS). A revision of an original GRADE criterion was only made if it could be methodologically justified, this means it was supported by statistical/epidemiological reasoning and could be supported by methodological articles. The results were gathered in a written document (TM, DP) and discussed in a group meeting with the whole project team. To facilitate the discussion, we illustrated the different challenges using example cases. If necessary, we refined the criteria until a consensus was reached. Note, no formalized consensus procedure was applied.

### Applications of specifications and adaptions

Subsequently, we performed a proof-of-concept application using a convenience sample including six systematic reviews focusing on safety known to us and for which we assumed that all specifications and adaptions would come into effect. These systematic reviews were not part of PRISCUS. In this phase, we checked our approach for any problems with a focus on inconsistencies and tendencies of overestimating the strength of evidence. In the proof-of-concept application, no reasons for revision of the criteria were recognized.

After this development phase, we used our approach for evidence syntheses prepared as basis for expert-rated recommendations on the PRISCUS-list. The GRADE assessment was performed by TM. All members of the team checked the final ratings for inconsistencies. In the pilot study, we assessed 51 outcomes for 19 clinical questions (see supplement) from 13 systematic reviews.

## Results

Table 1 shows the original GRADE criteria and our suggested adaptions. We explain and justify the adaptions in the following text. All domains/criteria not quoted in Table 1 were applied in the standard way as suggested by the GRADE working group.

**Table 1:** overview of adaptions

GRADE criteria	Original	Challenge	Specifications or adaptions
Study type/methodological quality	NRS start as “low quality” of evidence^a^	Data on harms from RCTs is often insufficient and thus it is advisable to consider NRS	NRS start as “high quality” of evidence if rated as low risk for confounding and selection bias
Imprecision (binary outcomes)	Usually, 95% of CIs of relative effects are used 95%CI overlaps decision threshold (e.g. null effect) → rating down one level 95%CI includes appreciable harm and benefit → rating down two levels	Harms are often rare events and rare in the included studies despite large sample sizes. In the case of rare events 95% CIs of relative effects can be misleading.	Imprecision is assessed based on absolute effects
Publication bias/missing results in the synthesis	Rating down for publication bias	For harms selective dissemination would result in underestimation of harms	Rating up for publication bias
Large magnitude of effect	Rating up if RR >2 (<0.5)	Harms are usually less affected by confounding by indication	Rating up if RR >1.67 (<0.60)
Originally not used	Subgroup effects	Harms are often subgroup-specific but analysis within subgroups is underpowered	Rating up if there is a statistically significant subgroup effect from a well-designed subgroup analysis

^a^When using the ROBINS-I tool NRS usually start as high quality of evidence and an adaption is not necessary

### Suggested specification/adaption “study type in relation to methodological quality”

NRS usually start as low quality of evidence because of the risk of confounding bias [13]. The approach can be also interpreted in the way that NRS are rated down two levels directly at the beginning because of risk for confounding. Consequently, all evidence on harms from NRS would start at “low quality” of evidence.

Recently a new approach was suggested when using the ROBINS-I tool for assessing the risk of bias of NRS by the GRADE working group [14]. It suggests that NRS start high but are regularly rated down by one or two levels because there is a risk of confounding and selection bias in NRS. In this sense, we adapted the original criteria. Precisely, we suggest NRS also start high irrespectively of the critical appraisal tool applied. Than all studies must be assessed for risk of confounding or participant selection bias. An adequate critical appraisal tool could be used or guide this assessment. As suggested for ROBINS-I, it might be useful to have a well-designed target trial in mind as the reference for evaluation. Furthermore, in the case that other bias in addition to confounding and selection bias are present it is possible to rate down three levels for study limitations (risk of bias) when using this approach [15]. Clearly, this adaption is not necessary when using ROBINS-I, and therefore this is the preferred tool for this task. However, the suggested adaption has the advantage that it can be applied when another tool for assessing risk of bias/methodological quality (e.g. because of limited resources) or already existing systematic reviews that apply other tools are used for assessing NRS. The approach appears reasonable for two reasons. First, confounding by indication is usually a minor issue for assessing harms compared to assessing effectiveness. In particular, rare harms and harms that are not obviously related to the intervention would mean that the effect is biased towards the null [16]. Second, most tools for assessing NRS consider cofounding and selection bias [17, 18]. Therefore, for most tools it is not necessary to start the assessment at low quality of evidence. An advantage that comes along with this approach is that double counting of confounding and selection bias is avoided, which exists when NRS start low and the applied quality assessment tool covers confounding or selection bias.

### Application example “study type in relation to methodological quality”

In our review on safety of tramadol in older people all evidence on falls risk was observational and consequently would have started at low quality of evidence, when assessed in the standard way. Based on the methodological quality assessment, the outcome was rated-down one level for confounding bias and one level for imprecision, which would have resulted in a very low quality of evidence rating because of double counting confounding and selection bias. Noticeable, in this case even only one criterion for rating-down would have led to a very low rating. In contrast, if assessed in the adapted way the evidence starts high and rating-down two levels resulted in a low quality of evidence rating.

### Suggested specification/adaption “imprecision”

Precision of an effect is usually assessed based on the 95% CI [19]. However, in the case of rare events this might be misleading. The GRADE working group suggests using absolute effects for very low event rates, whereby “very low” is not defined, instead. Harms, in particular severe harms are often rare. In addition, no fixed threshold exists at which the 95%CI of the relative effect should be preferred over the 95%CI of the absolute effect. Our preliminary suggestion is to use the absolute effects for assessing harms in general. Important to note, the 95%CI of the absolute effect is usually not reported and must be re-calculated. Furthermore, in the case of harms, it is especially important to use prevalence or incidence data, which is applicable to the target population (e.g. older people) because absolute effects often vary between sub-groups (e.g. falls in older people). Thus, for this purpose external data (not from the included studies) for calculating the control group risk using external data might be preferable.

### Application example “imprecision”

In a systematic review of artemethere lumefantrine versus Amodiaquine plus sulfadoxinee pyrimethamine for treating uncomplicated malaria the authors found 34 severe adverse events (SAEs) and calculated a risk ratio (RR) of 1.08 (95%-KI 0.56 to 2.08). The SAEs were observed in in >2.700 participants resulting in an absolute risk difference of 1% with (95%CI 0.6% less to 1.4% more) [19]. The example illustrates that although the 95%CIs of the RR suggest rating down two levels, the lower 95CI (possible avoidance of SAEs) of the absolute effect suggest rating down at most one level.

### Suggested specification/adaption “publication bias”

The quality of evidence is usually rated down one level when publication bias is detected [20]. The criteria for considering rating down because of publication bias are mainly small industry sponsored studies, or an asymmetric funnel-plot. Noticeable, a meta-epidemiological study found many clinical questions, which were suspicious for selective dissemination of safety outcomes [21]. In contrast to benefit outcomes, where publication bias would lead to an overestimation of the benefit because small (or in different direction) treatment effects are not available, missing safety results would result in an underestimation of harms because large effects for harms are not available [22]. Therefore, we suggest that authors may consider rating-up the quality of evidence, when there is strong suspicion for publication bias.

### Application example “publication bias”

In one of our reviews that was performed to inform experts on oral anticoagulants in older people all studies were industry sponsored, the expected harms were not reported for all studies (e.g. all bleeding events), or harms were grouped uncommonly (e.g. clinical relevant non-major bleeding), and the funnel-plot was slightly asymmetric. Therefore, we rated-up the quality of evidence for bleeding one level, from low to moderate.

### Suggested specification/adaption “large magnitude of effect”

The evidence usually can be rated-up by one level if the magnitude of the effect is large, whereby large is defined as an RR of >2 (<0.5). This threshold is based on the assumption that confounding alone is unlikely to cause such an large effect [23]. This threshold was determined based on a modelling study that was informed by older observational studies, which applied no methods to adjust for confounding [24]. Newer studies suggest that in the case the analyses of observational studies is adjusted for relevant confounders, it is unlikely that unmeasured and residual confounding alone lead to large or very large effects [25, 26]. Additionally, as mentioned above, harm outcomes are regularly less affected by bias by indication than effectiveness outcomes [16]. For these reasons, we suggest to consider rating-up harm outcomes one level if the RR is >1.67 (<0.60) for evidence based on unbiased and sufficiently precise NRS [25]. Moreover, we suggest to consider rating-up two levels if the RR is larger than 10 (<0.1) in NRS that are affected by confounding because previous studies suggest that such a large effect is unlikely to be caused by confounding alone [27].

### Application example “large magnitude of effect”

In our review on proton pump inhibitors compared to no proton pump inhibitors, we found a RR of 1.97 (95%CI 1.44 to 2.70) for dementia. The risk of bias for this estimate was low. Therefore, we rated-up the quality of evidence one level, from low to moderate, which would not have been done if applied in the original way.

### Suggested specification/adaption “subgroup-effects”

Risk of experiencing harms often varies between subgroups [28]. Therefore, exploring heterogeneity is in particular important when assessing harms. The GRADE guidance only considers subgroup-analyses to explore inconsistency between studies [29]. However, a statistically significant test of heterogeneity not only suggests that the effects are different between different groups and consequently should be considered separately but also comprises information on the certainty of effects. If there is a statistically significant sub-group effect, one could be more confident that there is an effect in this subgroup. Similar to a dose-response relationship, this is in particular true if the subgroup effect increases/decreases with increasing/decreasing level of the subgroup variable (e.g. the risk of experience harms increases with age). We suggest that when a well performed subgroup analysis (criteria for reliable subgroup analysis see for example [30]) indicates a larger effect in a subgroup, the quality of evidence in this subgroup might be rated up one level, in particular if the subgroup effect is level dependent [30].

### Application example “subgroup-effects”

In our review on oral anticoagulants in older people we extracted data from well performed (e.g. pre-specified, based on a test for interaction) within study subgroup-analyses for major bleeding. Most of these suggested that the risk of experiencing major bleeding increases with increasing age and that the risk of major bleeding is in particularly high in the very older people. For that reason, we rated-up the quality of evidence one level (from low to moderate) for bleeding risk in the very older people.

## Results of pilot testing

We found RCTs (only older people or subgroups analyses of older people) for only 9 of the 19 clinical questions. Each of the proposed adaptions was applied. Nevertheless, the ratings were well balanced. We rated 14 outcomes as high quality, 7 as moderate quality, 17 as low quality and 13 as very low quality. As expected, most “high methodological quality” ratings were made for clinical questions for which RCTs were available.

## Discussion and conclusion

A review of PIM-lists found that the applied methods were very heterogeneous and a look in these PIM-lists revealed that almost none appears to apply formalized methods to assess the quality of the underlying body of evidence for recommendations using formalized systems such as GRADE as it is standard for preparing clinical practice guidelines [3]. Only the last update of the Beers list used a joint assessment combining features of GRADE and the former recommendations developed by the Clinical Guidelines Committee of the American College of Physicians [31]. Although of interest, this work lacks a detailed methodological description, not enabling applying this methodological approach in another context. The reasons for the lack is probably that until now no specific system for assessing the quality of the body of evidence for evidence synthesizes focusing on harms such as for PIM-lists exists and the tools developed for clinical practice guidelines have some limitations because of the challenges that are common when considering safety (e.g. including NRS, rare events). As far as we know until now there neither is an approach to overcome these challenges nor is this problem discussed in the methodological literature. In this work, we propose some adaptions to or specify the application of the GRADE criteria when assessing safety outcomes in systematic reviews to inform a PIM-list.

The initial results of the application for preparing a PIM-list suggests that the ratings were quite well balanced. There were neither floor-effects (excessive number of low and very low ratings) nor ceiling effects (excessive number of high ratings), but the different methodological quality of the safety outcomes seems to be well reflected. The adaptions might have the potential to overcome some of the challenges when grading the methodological quality of harms and thus may be helpful for producers of evidence syntheses considering safety (e.g. literature for creating PIM-lists, systematic reviews on drug safety after approval). Although the adaptions were developed for evidence syntheses focusing on drug safety, we think that the adaptions might also be useful for evidence syntheses in general (i.e. all evidence syntheses considering benefits and harms) because basically all systematic reviews considering harms face the same challenges when grading the quality of evidence. Future research is desirable for developing refined GRADE guidance for evidence syntheses on harms.

The adaptions were not developed in a GRADE working group because the timeline of the project required that the evidence syntheses start immediately. Therefore, the suggested adaptions should only be regarded as a first step for stimulating further discussion and development of guidelines specifically for grading the quality of evidence on safety. This is in particular true for the suggestion that all NRS start high irrespective of the applied critical appraisal tool. Here, further research is needed that evaluates the impact of using different critical appraisal tools on the GRADE ratings. Likewise, the cut-off for (very) large effects remains controversial and require further evaluation [32].

## Supplementary Information

**Additional file 1.**

## Data Availability

Not applicable.

## Abbreviations

AE: Adverse event
NRS: No-randomized study
PIM: Potentially inappropriate medication
RCT: Randomized controlled trial
SAE: Serious adverse event
