# Supplementary material for: Assessing the quality of evidence on safety: specifications for application and suggestions for adaptions of the GRADE-criteria in the context of preparing a list of potentially inappropriate medications for older adults
Source: BMC Med Res Methodol. 2022 Aug 30;22:234. doi: 10.1186/s12874-022-01715-5 (PMC9426023; doi:10.1186/s12874-022-01715-5)
Supplement: Supplementary file 1 — Additional file 1. [file 12874_2022_1715_MOESM1_ESM.docx]

**Drug (if applicable with condition or outcome)**

1. proton pump inhibitors
2. benzodiazepines
3. alpha blockers
4. ginkgo biloba
5. anticholinergics for overactive bladder und beta-3- adrenoceptor agonist
6. Imidazolinrezeptoragonisten
7. tramadol
8. sulfonylurea
9. Z-drug
10. pregabalin
11. aldosterone antagonists
12. steroids for hip fracture
13. 2nd gen antipsychotics (SAEs)
14. 2nd gen antipsychotics (mortality)
15. antibiotics for UTI
16. laxatives iatrogenic (falls)
17. antihistamines (falls and fractures)
18. atypical antipsychotics for BPSD
19. antidepressants in 65 and older
